# Compressed Sensing Inspired Image Reconstruction from Overlapped Projections

**DOI:** 10.1155/2010/284073

**Published:** 2010-06-22

**Authors:** Lin Yang, Yang Lu, Ge Wang

**Affiliations:** ^1^Biomedical Imaging Division, VT-WFU School of Biomedical Engineering and Sciences, Virginia Tech, Blacksburg, VA 24061, USA; ^2^Department of Electrical and Computer Engineering, The Cooper Union for the Advancement of Science and Art, New York, NY 10003, USA; ^3^Department of Biomedical Engineering, Shanghai Jiao Tong University, Shanghai 200240, China

## Abstract

The key idea discussed in this paper is to reconstruct an image from overlapped projections so that the data acquisition process can be shortened while the image quality remains essentially uncompromised. To perform image reconstruction from overlapped projections, the conventional reconstruction approach (e.g., filtered backprojection (FBP) algorithms) cannot be directly used because of two problems. First, overlapped projections represent an imaging system in terms of summed exponentials, which cannot be transformed into a linear form. Second, the overlapped measurement carries less information than the traditional line integrals. To meet these challenges, we propose a compressive sensing-(CS-) based iterative algorithm for reconstruction from overlapped data. This algorithm starts with a good initial guess, relies on adaptive linearization, and minimizes the total variation (TV). Then, we demonstrated the feasibility of this algorithm in numerical tests.

## 1. Introduction

**Figure 1:**
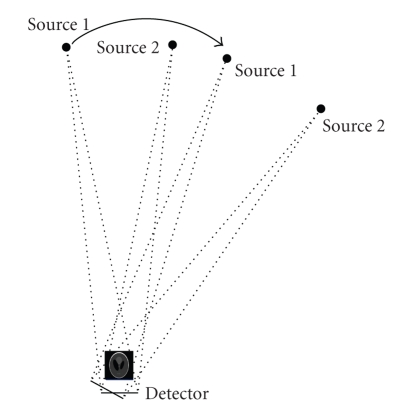
Imaging geometry for collection of overlapped projections from two X-ray sources.

The popular CT scheme takes projection data from an X-ray source being scanned along a trajectory and reconstructs an image from these data that are essentially line integrals through an object. In real-world applications, higher temporal resolution has been constantly pursued, such as for dynamic medical CT, micro-, and nano-CT. The multisource scanning mode is well known to improve temporal resolution but the data acquisition and field of view are seriously restricted to avoid overlapped projections, such as in the case of the classic dynamic spatial reconstructor (DSR). As shown in Figure 1, here we consider reconstructing an image from overlapped projections so that a new dimension of freedom can be offered to design novel CT architectures.

In the overlapped projection geometry, two (or more) sources, A and B, emit X-rays simultaneously through an object to be reconstructed from various orientations. As a result, the resultant X-ray projections are overlapped onto the same detector array. The overlapped projections use the same detector array at the same time but complicate the imaging model. To perform image reconstruction from overlapped projections, the conventional reconstruction approach (e.g., filtered backprojection (FBP) algorithms) cannot be directly used because of the two problems. First, overlapped projections represent an imaging system in terms of summed exponentials, which cannot be transformed into a linear form, since the X-ray intensity through an object follows an exponential decaying function. Second, overlapped measurement carries less information than the traditional line integrals, due to the additional uncertainty from mixing two ray sums, leading to an underdetermined imaging system.

Compressive sensing (CS) is a new technique being rapidly developed over the past years [1, 2]. It has been shown that if a vector **x** contains at most *S* nonzero elements and there are *K* random measurements of **x** such that *K* ≥ *C* · *S* · log (*N*), where *C* is a constant and *N* is the dimension of **x**, then minimizing the L-1 norm of **x** reconstructs **x** perfectly with an overwhelming probability. Inspired by its success in signal recovery, we propose a compressive sensing- (CS-) inspired iterative algorithm for reconstruction from overlapped data. This algorithm starts with a good initial guess, relies on adaptive linearization, and minimizes the total variation (TV). Then, we demonstrated the feasibility of this algorithm in numerical tests. The rest of this paper is organized as follows: in Section 2 we describe our algorithms for data synthesis and image reconstruction, in Section 3 we report numerical results under different conditions, and in Section 4 we discuss relevant issues and conclude the paper.

## 2. Methodology

An image *f* can be discretized into a *W* by *H* matrix, which can be represented as a vector **f** of length *n* = *W* · *H*. Let *N*_src_ denote the total number of X-ray sources (for a dual-source system, *N*_src_ = 2, which is the focused case in this paper), *N*_bin_ the total number of linear or area detector bins, and *N*_rot_ the total number of view angles. Then, the sampling process will yield *N*_bin_ · *N*_rot_ overlapped data. Since the X-ray attenuation is governed by an exponential decaying function, the overlapped projection data can be expressed as

(1) $$\begin{array}{cc} p & =\left[\begin{array}{c} p_{1,1}\\ p_{2,1}\\ \vdots \\ p_{N_{\text{bin}},1}\\ \vdots \\ p_{1,N_{\text{rot}}}\\ \vdots \\ p_{N_{\text{bin}},N_{\text{rot}}} \end{array}\right]=\exp\;\left(-M_{1}f\right)+\exp\;\left(-M_{2}f\right)\\ & \;+\cdots +\exp\;\left(-M_{N_{\text{src}}}f\right)\\ & =\left[\begin{array}{c} \exp\;\left(-M_{1,1,1}f\right)\\ \exp\;\left(-M_{1,2,1}f\right)\\ \vdots \\ \exp\;\left(-M_{1,N_{\text{bin}},1}f\right)\\ \exp\;\left(-M_{1,1,2}f\right)\\ \vdots \\ \exp\;\left(-M_{1,N_{\text{bin}},2}f\right)\\ \vdots \\ \exp\;\left(-M_{1,1,N_{\text{rot}}}f\right)\\ \vdots \\ \begin{array}{c} \exp\;\left(-M_{1,N_{\text{bin}},N_{\text{rot}}}f\right) \end{array} \end{array}\right]+\left[\begin{array}{c} \exp\;\left(-M_{2,1,1}f\right)\\ \exp\;\left(-M_{2,2,1}f\right)\\ \vdots \\ \exp\;\left(-M_{2,N_{\text{bin}},1}f\right)\\ \exp\;\left(-M_{2,1,2}f\right)\\ \vdots \\ \exp\;\left(-M_{2,N_{\text{bin}},2}f\right)\\ \vdots \\ \exp\;\left(-M_{2,1,N_{\text{rot}}}f\right)\\ \vdots \\ \begin{array}{c} \exp\;\left(-M_{2,N_{\text{bin}},N_{\text{rot}}}f\right) \end{array} \end{array}\right]\\ & \;+\cdots +\left[\begin{array}{c} \exp\;\left(-M_{N_{\text{src}},1,1}f\right)\\ \exp\;\left(-M_{N_{\text{src}},2,1}f\right)\\ \vdots \\ \exp\;\left(-M_{N_{\text{src}},N_{\text{bin}},1}f\right)\\ \exp\;\left(-M_{N_{\text{src}},1,2}f\right)\\ \vdots \\ \exp\;\left(-M_{N_{\text{src}},N_{\text{bin}},2}f\right)\\ \vdots \\ \exp\;\left(-M_{N_{\text{src}},1,N_{\text{rot}}}f\right)\\ \vdots \\ \begin{array}{c} \exp\;\left(-M_{N_{\text{src}},N_{\text{bin}},N_{\text{rot}}}f\right) \end{array} \end{array}\right], \end{array}$$

where **p** is an *N*_bin_ · *N*_rot_ by 1 vector whose element *p*_*m*,*r*_, *m* ∈ {1,2,…, *N*_bin_} and *r* ∈ {1,2,…, *N*_rot_}, is the overlapped projection datum detected by the *m*th detector bin at the *r*th view angle, *M*_*l*_ denotes the system matrix for the *l*th source, and *l* ∈ {1,2,…, *N*_src_}. The 1 by *n* row vector **M**_*l*,*m*,*r*_ is the X-ray intersection length vector from the *l*th source to the *m*th detector bin at the *r*th view angle. The *k*th entry of **M**_*l*,*m*,*r*_ is obtained by calculating the intersection length of the involved X-ray through the *k*th pixel of **f**, which corresponds to the indices *l*, *m*, and *r*. The system matrix *M* and overlapped projection data can be readily computed in different ways. For example, in reference to [3], we have Algorithm 1 to generate projection data.

Now, the key issue is how to reconstruct an image from overlapped projection data. To alleviate the underdetermined measurement due to the overlapped nature of projection data, the compressed sensing (CS) principles are employed in our reconstruction process. To utilize the sparsity of an underlying image, it is first transformed into a gradient counterpart, and then the L-1 norm of the gradient, which is known as the total variation (TV), is minimized, subject to the overlapped projection data. The entire reconstruction process can therefore be casted into a constrained nonlinear optimization problem:

 
*Minimize: TV of* **f** *subject to* **p** = exp (*M*_1_ **f**) + exp (*M*_2_ **f**)* and other constraints (such as intensity ranges and object features)*

Clearly, there are various ways to solve the above constrained TV minimization problem. In the CS field, a projection onto convex sets (POCS) and gradient descent search approach has been successfully used to solve this type of MRI and CT imaging problems [4–9]. POCS takes advantage of the fact that the linear constraints are hyper-planes in the *n*-dimensional space so that a closed form solution for the projection onto these hyper-planes can be derived. Such an algorithm works well in the single source geometry, because raw projection data can be processed into line integrals.

However, in the case of overlapped projections from two sources, the constraint equations, which are the sums of two exponentials, cannot be transformed into a linear form. Therefore, a different approach is needed. Our solution is to make a good initial guess, such as a low-resolution CT image first. This blurry image will serve as a starting point, and the difference between this initial reference and the actual image will be iteratively updated, and at the same time the current guess will be also updated. Since the difference is assumed to be small, we can perform a Taylor series expansion to linearize the imaging system by omitting high-order terms. Then, we can apply the POCS-gradient algorithm on this linearly approximated system iteratively.

Mathematically, let us denote **f** = **g** + **d** **f**, where **f** is the original image, **g** the blurry image, and **d** **f** the difference between **f** and **g**. Then, we have

(2) $$\begin{array}{cc} p & =\exp\;\left(-M_{1}\cdot f\right)+\exp\;\left(-M_{2}\cdot f\right)\\ & =\exp\;\left(-M_{1}\cdot g\right)\exp\;\left(-M_{1}\cdot df\right)\\ & \;+\exp\;\left(-M_{2}\cdot g\right)\exp\;\left(-M_{2}\cdot df\right)\\ & =\exp\;\left(-M_{1}\cdot g\right)\left(\overset{\infty }{\underset{n=0}{\sum }}\frac{{\left(-M_{1}\cdot df\right)}^{n}}{n!}\right)\\ & \;+\exp\;\left(-M_{2}\cdot g\right)\left(\overset{\infty }{\underset{n=0}{\sum }}\frac{{\left(-M_{2}\cdot df\right)}^{n}}{n!}\right)\\ & \approx \exp\;\left(-M_{1}\cdot g\right)\left(1+\left(-M_{1}\cdot df\right)\right)\\ & \;+\exp\;\left(-M_{2}\cdot g\right)\left(1+\left(-M_{2}\cdot df\right)\right)\\ & =\exp\;\left(-M_{1}\cdot g\right)-\exp\;\left(-M_{1}\cdot g\right)M_{1}\cdot df+\exp\;\left(-M_{2}\cdot g\right)\\ & \;-\exp\;\left(-M_{2}\cdot g\right)M_{2}\cdot df. \end{array}$$

That is,

(3) $$\begin{array}{cc} & \left[\exp\;\left(-M_{1}\cdot g\right)M_{1}+\exp\;\left(-M_{2}\cdot g\right)M_{2}\right]\cdot df\\ & \;\;=-p+\exp\;\left(-M_{1}\cdot g\right)+\exp\;\left(-M_{2}\cdot g\right). \end{array}$$

Then, we have the approximate system

(4) $$M_{\text{new}}df=p_{\text{new}},$$

where

(5) $$\begin{array}{cc} M_{\text{new}} & =\exp\;\left(-M_{1}\cdot g\right)M_{1}+\exp\;\left(-M_{2}\cdot g\right)M_{2},\\ p_{\text{new}} & =-p+\exp\;\left(-M_{1}\cdot g\right)+\exp\;\left(-M_{2}\cdot g\right). \end{array}$$

The above approximate system is linear with respect to **d** **f**. This linearity allows us to perform POCS on **d** **f**. To perform the gradient descent search on the TV of **g** + **d** **f**, we compute the gradient of the TV explicitly in the image domain, for example, using the formulas described in [8]. After the linearization with respect to **d** **f** and the formulation of the TV gradient, we can apply the POCS-gradient algorithm to estimate **d** **f**. Note that such a reconstructed image **g** + **d** **f** can be used as a new guess in the POCS-gradient process until a satisfactory reconstruction is achieved, as summarized in Algorithm 2.

## 3. Numerical Experiments

To demonstrate the feasibility of our proposed algorithm for image reconstruction from overlapped projections, we developed a program in MATLAB, and implemented the traditional algebraic reconstruction technique (ART) for comparison. A Modified 2D Shepp-Logan phantom (Table 2) was scaled into a 5 cm by 5 cm square and discretized into a 256 × 256 matrix. The phantom was centered at the origin of the reconstruction coordinate system. A circular scanning trajectory of radius 121.66 cm was assumed with the two sources initially located at (−20 cm, 120 cm) and (20 cm, 120 cm), respectively. A 14 cm linear detector array was positioned opposite to the sources and 5 cm below the phantom with a distance of 131.53 cm from each of the sources. Gaussian white noise was drawn from the normal distribution *N*(0, 0.005) and added to ideal projection data during the sampling stage. The scanning geometry is illustrated in Figure 1. The other parameters are listed in Table 1.

**Figure 2:**
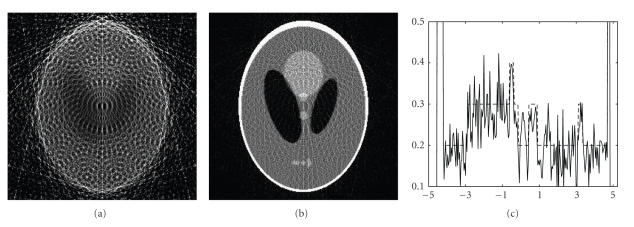
Reconstructed images of the Shepp-Logan phantom in the first test. (a) A reconstruction using ART, (b) a reconstruction using IROP, and (c) the profiles along the central vertical line of the phantom, where the dotted and solid lines are for the phantom and the IROP reconstruction, respectively (the display window: [0,0.5]).

**Figure 3:**
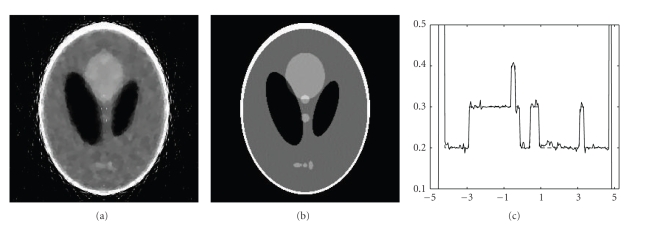
Reconstructed images of the Shepp-Logan phantom in the second test. (a) A reconstruction using ART, (b) a reconstruction using IROP, and (c) the profiles along the central vertical line of the phantom, where the dotted and solid lines are for the phantom and the IROP reconstruction, respectively (the display window: [0,0.5]).

**Figure 4:**
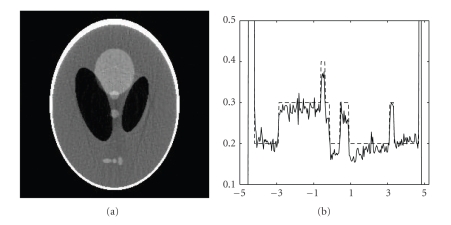
Reconstructed images of the Shepp-Logan phantom in the third test. (a) A reconstruction using IROP with a constant initial guess, and (b) the profiles along the central vertical line of the phantom, where the dotted and solid lines are for the phantom and the IROP reconstruction, respectively (the display window: [0,0.5]).

We performed both ART and IROP reconstructions under these conditions, with blurry and constant initial guesses. Representative results are in Figures 2, 3, and 4. It has been observed in our simulation that our IROP algorithm would work well if the initial guess resembles the ideal image through a moderate blurring process. Actually, in the first test the blurry images were obtained by blurring a low-quality ART image reconstructed under a severely under-sampling condition with only *N*_bin_ × *N*_rot_ = 15 × 50 = 750 measurements to reconstruct 256 × 256 = 65536 pixels. In the second test, more measurements were made in the single source scan, and the IROP reconstruction became better. Also, the IROP reconstruction turned to be smoother than the corresponding ART images, indicating that compressed sensing (CS) is more effective than ART in suppressing image noise. In the last test, we reconstructed an IROP image with a constant initial guess (a zero image). The reconstructed image can be further improved if we use more iterations. 

**Figure 5:**
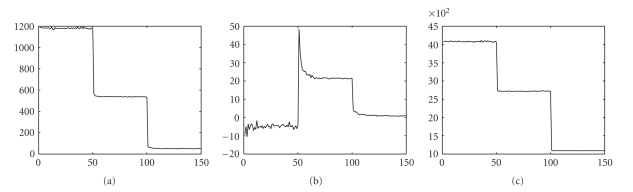
Convergence plots for (a) Test 1, (b) Test 2, and (c) Test 3, with 3 linearization steps and 50 iterations after each linearization.

**Figure 6:**
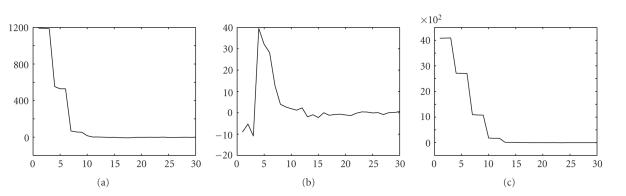
Convergence plots for (a) Test 1, (b) Test 2, and (c) Test 3, with 10 linearization steps and 3 iterations after each linearization step.

To investigate the convergence of IROP, we first introduce an evaluation metric *δ*(*n*), which is defined as the sum of the component values in the error vector **d** **f** at *n*th iteration:

(6) $$\delta \left(n\right)=\overset{256^{2}}{\underset{i=1}{\sum }}df_{i}\left(n\right),$$

where the subscript *i* denotes the *i*th pixel component in the error vector **d** **f**. We then plotted *δ*(*n*) for every iteration. The results for each of the three tests are shown in Figure 5. There are mainly three important observations from the convergence plots. First, the big jumps at multiples of *N*_itr_ indicate that linearization step played an important role in the convergence of IROP. Second, as IROP goes through more iterations, *δ*(*n*) approaches zero, showing that IROP effectively reduces the differences between the original and the reconstructed images. Finally, the smaller values for *δ*(*n*) in test 2 show that a good initial guess can lead to better reconstruction quality. Figure 6 shows another 3 plots obtained with fewer iterations and more linearizations. It is observed that the error between the reconstructed image and the true image was reduced dramatically immediately after each new linearization. After 3 or 4 linearization processes, the convergence curve became stable and smooth. After that, if we increased the number of iterative steps in each linearization process, the image quality would be improved only slowly. Hence, to balance image quality and computational time, a good solution is for the method to use a limited number of iterative steps after each earlier linearization process and perform a sufficiently large number of iterative steps after the final linearization, for example, after 3 or 4 linearization processes.

## 4. Discussions and Conclusion

The primary advantage of the IROP scheme is to improve the data acquisition speed. In one exemplary application, we can assume that the two sources are fairly close so that the detector collimation can work effectively for both the sources. If a good number of sources are used, scattering effects could be a concern. In that case, scattering correction may be needed using hardware (such as some degree of multiplexing) and/or software (such as model- or image-based compensation) methods [10–13].

In Algorithm 2, the key for the linearization to be successful is to have a good initial guess. It is underlined that it is practical to have such a good guess. For example, in multiresolution CT studies, a low-resolution image serves as a guess naturally. Also, in dynamic CT studies, an initial image represents a good guess to subsequent images. When we have a cluster of computers, we may use multiple random initial guesses to search for a more accurate and stable reconstruction. Furthermore, Algorithm 2 may be adapted into an evolutionary scheme.

The implementation of Algorithm 2 can be improved in several ways. To reduce the smoothing artifact, one can reduce the number of gradient descent iterations or the step size. Other algorithmic parameters could also be tuned for a specific type of applications. Most importantly, the computational structure of Algorithm 2 is really based on simple heuristics and does not reflect all the constraints and requirements in a well-integrated and optimized fashion. It is possible and desirable to design brand new algorithms that involve less parameters and have better properties. 

A theoretical analysis on the convergence of the IROP scheme has not been performed yet but we hypothesize that the global convergence can be established if a guess is appropriately chosen, as numerically shown in the preceding section. Actually, the IROP problem is much better posed than many well-known inverse problems such as diffuse optical tomography (DOT) [14]. In IROP with two sources, each datum reflects information from two lines. In DOT, each measure is related to a random zigzag trajectory. Thus, it is not surprising to see better results with IROP than that with DOT. When we have infinitely many sources along a line, we have a line-source imaging geometry, which has been studied by Bharkhada et al. [15] and still yields better results than DOT reconstruction [15]. 

Since the IROP scheme mixes line integrals pairwise, the IROP problem may lead to an underdetermined system of measurement equations, especially when the number of samples is not sufficiently large for ultrafast imaging performance. To address this issue, we have implemented the CS principles in Algorithm 2 by minimizing the TV. CS is a contemporary technique for solving an underdetermined system of linear equations, whose solution is known to be sparse. The main idea is to minimize cardinality, or equivalently to minimize the TV in many cases. In the context of IROP, an image itself is usually not sparse, but it can be sparsified in a transformed domain such as the gradient transform, and then we can apply the L-1 norm minimization in the transformed domain subject to the projection data constraints for good reconstructions, as numerically shown in the preceding section. 

It is emphasized that our IROP approach can be extended to multiple other imaging scenarios. For example, in transmission ultrasound imaging, we can use multiple ultrasound sources and a single array of detectors (transducers). This may be also related to the area of signal unmixing. The common task would be to unravel an underlying signal or image from mixed measures. There seem good research opportunities along this direction.

In conclusion, we have proposed the idea to perform image reconstruction from overlapped projection data and formulated a CS-based iterative algorithm for this new imaging problem. Our IROP algorithm starts with a good initial guess, relies on adaptive linearization, and minimizes the TV. Also, we have demonstrated the feasibility of this algorithm in numerical simulation. Further research is being performed to characterize and improve our IROP approach.

## Figures and Tables

**Algorithm 1:**
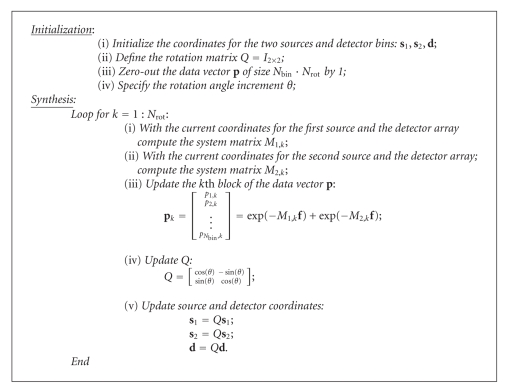
Synthesis of overlapped projection data.

**Algorithm 2:**
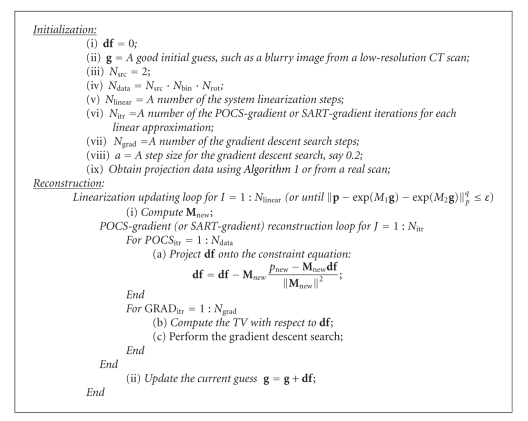
Image Reconstruction from overlapped projections (IROP).

**Table 1:** Parameters used in the numerical tests.

	*Test 1*		*Test 2*		*Test 3*	
	ART: Single Source	IROP: Two Source	ART: Single Source	IROP: Two Source	ART: Single Source	IROP: Two Source
*N*_rot_	15	150	30	150	NA	150
*N*_bin_	50	500	100	500	NA	500
*N*_linear_	NA	3	NA	3	NA	3
*N*_itr_	50	50	50	50	NA	50
*N*_grad_	NA	5	NA	5	NA	5

**Table 2:** Parameters of the 2D modified Shepp-Logan phantom.

Axis length (*a*,*b*)	Center (*x*,*y*)	Angle (*θ*)	Density
(0.690, 0.92)	(0, 0)	0	1.0
(0.6624, 0.874)	(0, −0.0184)	0	−0.8
(0.11, 0.31)	(0.22, 0)	−18	−0.2
(0.16, 0.41)	(−0.22, 0)	18	−0.2
(0.21, 0.25)	(0, 0.35)	0	0.1
(0.046, 0.046)	(0, 0.1)	0	0.1
(0.046, 0.046)	(0, −0.01)	0	0.1
(0.046, 0.023)	(−0.08, −0.605)	0	0.1
(0.023, 0.023)	(0, −0.606)	0	0.1
(0.023, 0.046)	(0.06, −0.606)	0	0.1
